# 11-(4-Methyl­phen­yl)-8,9-dihydro-7*H*-benzo[*f*]cyclo­penta­[*b*]quinolin-10(11*H*)-one

**DOI:** 10.1107/S1600536812035659

**Published:** 2012-08-23

**Authors:** Cao Yang

**Affiliations:** a The First Affiliated Hospital of Nanjing Medical University, Nanjing 210029, People’s Republic of China

## Abstract

In the title compound, C_23_H_19_NO, the naphthalene ring system and the cyclo­pent-2-enone ring exhibit planar conformations with maximum deviations of 0.034 (1) and 0.02 (1) Å, respectively. The 1,4-dihydro­pyridine ring adopts an envelope conformation with the C atom bearing the *p*-tolyl ring as the flap atom. Inter­molecular N—H⋯O hydrogen bonds and C—H⋯π inter­actions stabilize the crystal packing.

## Related literature

For the medicinal use of quinoline and fused quinoline derivatives, see: Audisio *et al.* (2012 ▶); Kurasawa *et al.* (2012 ▶); Pokhrel *et al.* (2012 ▶). For puckering parameters, see: Cremer & Pople (1975 ▶).
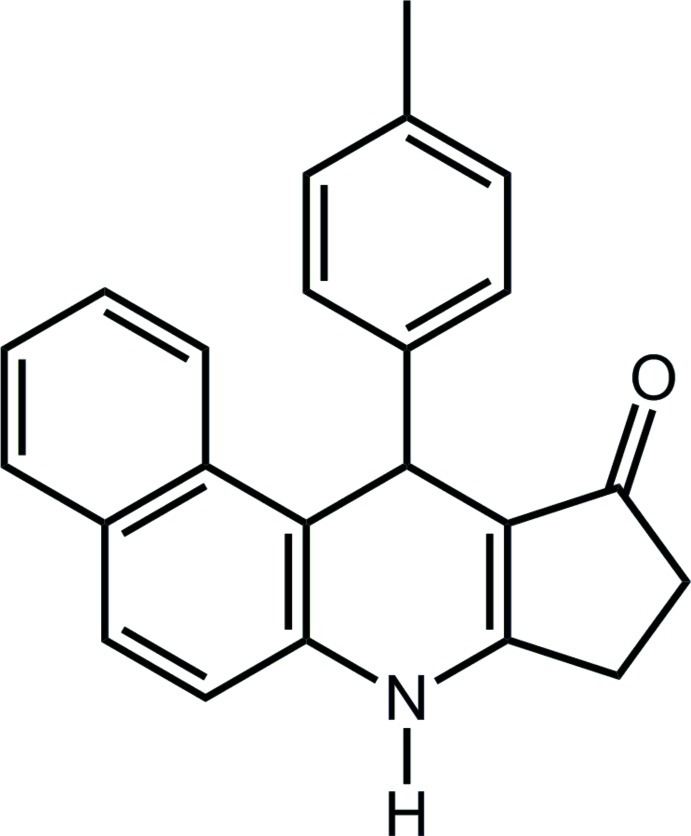


## Experimental

### 

#### Crystal data

C_23_H_19_NO
*M* *_r_* = 325.39
Monoclinic, 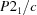

*a* = 8.727 (1) Å
*b* = 11.6820 (14) Å
*c* = 16.240 (2) Å
β = 98.938 (5)°
*V* = 1635.5 (3) Å^3^
*Z* = 4
Mo *K*α radiation
μ = 0.08 mm^−1^
*T* = 113 K
0.24 × 0.20 × 0.18 mm

#### Data collection

Rigaku Saturn724 CCD diffractometer
Absorption correction: multi-scan (*CrystalClearSM Expert*; Rigaku/MSC, 2009 ▶) *T*_min_ = 0.981, *T*_max_ = 0.986
15928 measured reflections
3899 independent reflections
2991 reflections with *I* > 2σ(*I*)
*R*_int_ = 0.031

#### Refinement

*R*[*F* ^2^ > 2σ(*F* ^2^)] = 0.044
*wR*(*F* ^2^) = 0.124
*S* = 1.07
3899 reflections
231 parameters
H atoms treated by a mixture of independent and constrained refinement
Δρ_max_ = 0.36 e Å^−3^
Δρ_min_ = −0.32 e Å^−3^

### 

Data collection: *CrystalClearSM Expert* (Rigaku/MSC, 2009 ▶); cell refinement: *CrystalClearSM Expert*; data reduction: *CrystalClearSM Expert*; program(s) used to solve structure: *SHELXS97* (Sheldrick, 2008 ▶); program(s) used to refine structure: *SHELXL97* (Sheldrick, 2008 ▶); molecular graphics: *SHELXTL* (Sheldrick, 2008 ▶); software used to prepare material for publication: *SHELXTL*.

## Supplementary Material

Crystal structure: contains datablock(s) I, global. DOI: 10.1107/S1600536812035659/hg5239sup1.cif

Structure factors: contains datablock(s) I. DOI: 10.1107/S1600536812035659/hg5239Isup2.hkl

Supplementary material file. DOI: 10.1107/S1600536812035659/hg5239Isup3.cml

Additional supplementary materials:  crystallographic information; 3D view; checkCIF report

## Figures and Tables

**Table 1:** Hydrogen-bond geometry (Å, °) *Cg*1 is the centroid of the C17–C22 ring.

*D*—H⋯*A*	*D*—H	H⋯*A*	*D*⋯*A*	*D*—H⋯*A*
N1—H1⋯O1^i^	0.902 (17)	1.983 (17)	2.8519 (13)	161.2 (14)
C15—H15⋯*Cg*1^ii^	0.95	3.00	3.8110 (15)	145

Symmetry codes: (i) 
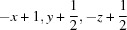
; (ii) 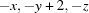.

**Fig. 1.**
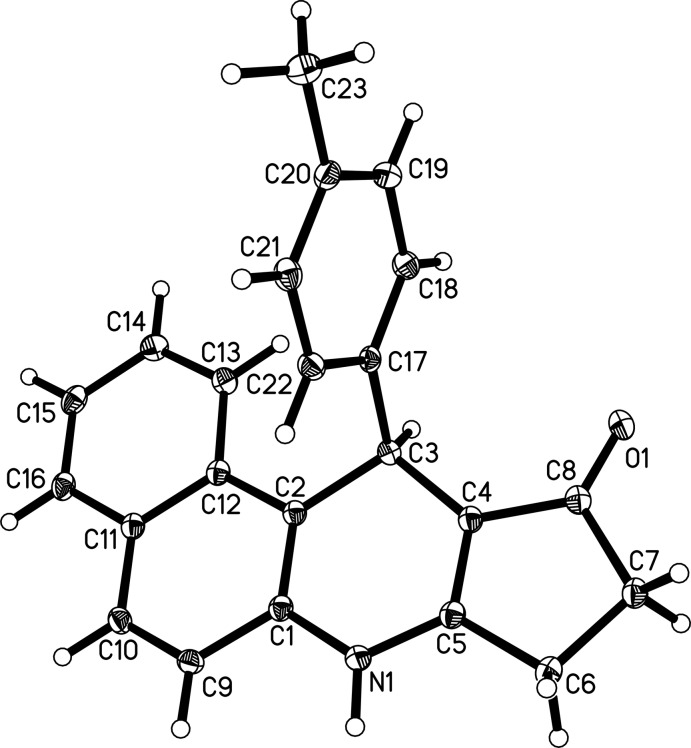
The structure of (I), showing 30% probability displacement ellipsoids and the atom-numbering scheme.

**Fig. 2.**
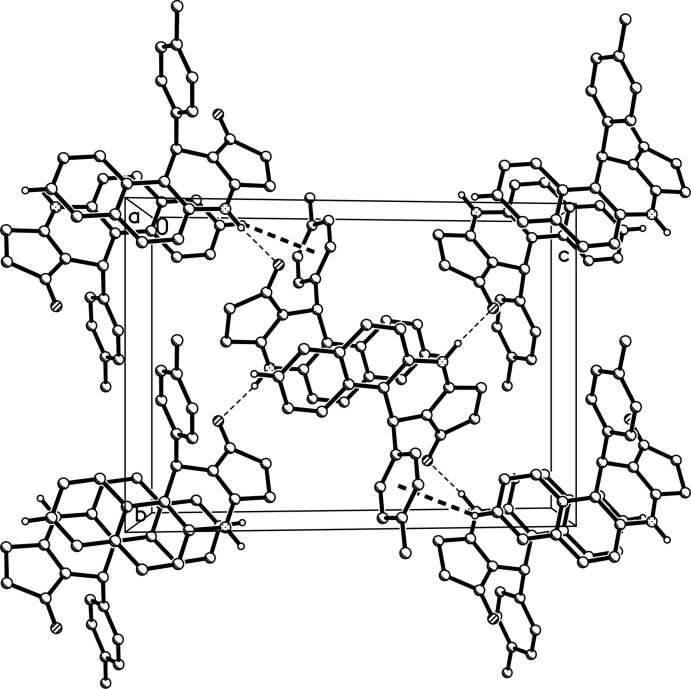
The packing diagram of (I), Hydrogen bond represented by the dashed line, Cg is the centroid of the ring of C17/C18/C19/C20/C21/C22, Hydrogen atoms not involved in Hydrogen bond were omitted for clarity.

## supplementary crystallographic information

### Comment

Many efforts have been devoted to the synthesis of quinoline and fused quinoline derivaties due to their broad spectrum of bioactivities including anti-norovirus activity (Pokhrel *et al.* 2012), antikinetoplastid activity (Audisio *et al.* 2012), antimalarial activity (Kurasawa *et al.* 2012). These work inspired us to assemble various types of fused quinoline skeletons and evaluate their bioactivities to study the relationship between structure and activity (SAR). Determination of the accurate structure is crucial to do SAR research. During our work on the efficient synthesis and biological study of benzo[*f*]cyclopenta[*b*]quinolins, the title compound (I) was isolated and its structure was determined by X-ray diffraction. Herein we report its crystal structure.

In the molecular structure (Fig. 1), the quinoline ring and cyclopent-2-enone ring adopts planar conformations. The atoms with maximal deviation from the the two ring are C9 and ing systr C5 with the distance of 0.034 (1)Å and 0.02 (1) Å, respectively. The dihydropyridine ring is in an envelope (or half boat) conformation, since C1/C2/N1/C4/C5 is coplanar with the largest deviation of 0.040 (1)Å(N1). However, the distance of C3 and the plane consisting of the rest five atoms is 0.212 (1)Å. Cremer & Pople puckering parameters analysis also confirms that the dihydropyridine ring adopts a envelope conformation (Cremer & Pople, 1975). Its puckering amplitude (Q), θ and φ are 0.156 (1)Å, 102.7 (4)° and 0.7 (5)°, respectively. The linkage between *p*-tolyl and dihydropyridine ring can be described by the torsion angle of C2—C3—C17—C22 (45.46 (15)°) and C4—C3—C17—C22(-76.38 (13)°) respectively. The crystal packing is stabilized by intermolecular N—H···O hydrogen bond and C—H··· π interactions (Fig. 2, Table 1).

### Experimental

In a dry 50 mL flask, cyclopentane-1,3-dione (0.10 g, 1 mmol), 4-methylbenzaldehyde (0.12 g, 1 mmol), naphthalen-2-amine (0.14 g,1 mmol), *p*-toluenesulfonic acid (0.2 g, 1 mmol) and water (10 mL) were mixed and then stirred at 373 K for 5 h. After completion of the reaction, as indicated by TLC, the solid product was collected by filtration and were purified by flash column chromatography (silica gel, mixtures of ethyl acetate / petroleum ether, 1:3, *v*/*v*) to afford the desired pure product. m. p.: 562-564 K; IR (KBr,ν, cm^-1^): 3165, 3080, 3013, 2962, 1666, 1632, 1584, 1522, 1468, 1397, 1269, 1239, 1220, 1012, 841, 806, 748, 688, 531; ^1^H NMR (400 MHz, DMSO-*d*_6_) (δ, ppm): 10.27 (s, 1H, NH), 7.80 (q, *J* = 8.4 Hz, 3H, ArH), 7.37-7.28 (m, 3H, ArH), 7.05 (d, *J* = 8.0 Hz, 2H, ArH), 6.94 (d, *J* = 8.0 Hz, 2H, ArH), 5.58 (s, 1H, CH), 2.71-2.63 (m, 2H, CH_2_), 2.34-2.20 (m, 2H, CH_2_), 2.14 (s, 3H, CH_3_); HRMS (ESI) m/z: Calcd. for C_23_H_19_NONa [M+Na]^+^ 348.1364, found: 348.1345.The single-crystal suitable for X-ray diffraction was obtained through slow evaporation of the ethanol solution of the title compound.

### Refinement

The hydrogen atom bonded to nitrogen atom was positioned from a Fourier difference map refined freely. All other H atoms were placed in calculated positions, with C—H = 0.95 Å, 0.98 Å, 0.99 Å or 1.00 Å and included in the final cycles of refinement using a riding model, with U_iso_(H) = 1.2U_eq_(parent atom).

### Crystal data

**Table:**

C_23_H_19_NO	*F*(000) = 688
*M**_r_* = 325.39	*D*_x_ = 1.321 Mg m^−^^3^
Monoclinic, *P*2_1_/*c*	Mo *K*α radiation, λ = 0.71075 Å
Hall symbol: -P 2ybc	Cell parameters from 5530 reflections
*a* = 8.727 (1) Å	θ = 1.7–27.9°
*b* = 11.6820 (14) Å	µ = 0.08 mm^−^^1^
*c* = 16.240 (2) Å	*T* = 113 K
β = 98.938 (5)°	Prism, colorless
*V* = 1635.5 (3) Å^3^	0.24 × 0.20 × 0.18 mm
*Z* = 4	

### Data collection

**Table:**

Rigaku Saturn724 CCD diffractometer	3899 independent reflections
Radiation source: rotating anode	2991 reflections with *I* > 2σ(*I*)
Multilayer monochromator	*R*_int_ = 0.031
Detector resolution: 14.222 pixels mm^-1^	θ_max_ = 27.9°, θ_min_ = 2.2°
ω scans	*h* = −11→11
Absorption correction: multi-scan (*CrystalClearSM Expert*; Rigaku/MSC, 2009)	*k* = −15→15
*T*_min_ = 0.981, *T*_max_ = 0.986	*l* = −21→19
15928 measured reflections	

### Refinement

**Table:**

Refinement on *F*^2^	Primary atom site location: structure-invariant direct methods
Least-squares matrix: full	Secondary atom site location: difference Fourier map
*R*[*F*^2^ > 2σ(*F*^2^)] = 0.044	Hydrogen site location: inferred from neighbouring sites
*wR*(*F*^2^) = 0.124	H atoms treated by a mixture of independent and constrained refinement
*S* = 1.07	*w* = 1/[σ^2^(*F*_o_^2^) + (0.0777*P*)^2^ + 0.041*P*] where *P* = (*F*_o_^2^ + 2*F*_c_^2^)/3
3899 reflections	(Δ/σ)_max_ < 0.001
231 parameters	Δρ_max_ = 0.36 e Å^−^^3^
0 restraints	Δρ_min_ = −0.32 e Å^−^^3^

### Special details

**Table:**

Geometry. All e.s.d.'s (except the e.s.d. in the dihedral angle between two l.s. planes) are estimated using the full covariance matrix. The cell e.s.d.'s are taken into account individually in the estimation of e.s.d.'s in distances, angles and torsion angles; correlations between e.s.d.'s in cell parameters are only used when they are defined by crystal symmetry. An approximate (isotropic) treatment of cell e.s.d.'s is used for estimating e.s.d.'s involving l.s. planes.
Refinement. Refinement of *F*^2^ against ALL reflections. The weighted *R*-factor *wR* and goodness of fit *S* are based on *F*^2^, conventional *R*-factors *R* are based on *F*, with *F* set to zero for negative *F*^2^. The threshold expression of *F*^2^ > σ(*F*^2^) is used only for calculating *R*-factors(gt) *etc*. and is not relevant to the choice of reflections for refinement. *R*-factors based on *F*^2^ are statistically about twice as large as those based on *F*, and *R*- factors based on ALL data will be even larger.

### Fractional atomic coordinates and isotropic or equivalent isotropic displacement parameters (Å^2^)

**Table:**

	*x*	*y*	*z*	*U*_iso_*/*U*_eq_	
O1	0.35410 (9)	0.68276 (7)	0.17550 (5)	0.0239 (2)	
N1	0.67402 (12)	1.00971 (9)	0.20388 (6)	0.0210 (2)	
C1	0.71537 (13)	1.01482 (10)	0.12380 (7)	0.0189 (3)	
C2	0.67622 (12)	0.92753 (9)	0.06669 (7)	0.0181 (2)	
C3	0.59399 (13)	0.81856 (9)	0.08934 (7)	0.0181 (3)	
H3	0.5039	0.8036	0.0446	0.022*	
C4	0.53334 (13)	0.83755 (10)	0.17008 (7)	0.0190 (3)	
C5	0.57787 (13)	0.92659 (10)	0.22198 (7)	0.0198 (3)	
C6	0.50911 (14)	0.92521 (10)	0.30110 (8)	0.0238 (3)	
H6A	0.4506	0.9964	0.3075	0.029*	
H6B	0.5903	0.9155	0.3504	0.029*	
C7	0.40108 (14)	0.82132 (10)	0.28894 (8)	0.0227 (3)	
H7A	0.4289	0.7654	0.3346	0.027*	
H7B	0.2919	0.8451	0.2879	0.027*	
C8	0.42339 (13)	0.76912 (10)	0.20551 (8)	0.0204 (3)	
C9	0.79777 (13)	1.11263 (10)	0.10358 (8)	0.0216 (3)	
H9	0.8258	1.1702	0.1445	0.026*	
C10	0.83712 (13)	1.12502 (10)	0.02645 (8)	0.0229 (3)	
H10	0.8904	1.1920	0.0134	0.027*	
C11	0.79946 (13)	1.03903 (10)	−0.03478 (8)	0.0199 (3)	
C12	0.72028 (13)	0.93887 (10)	−0.01424 (7)	0.0188 (2)	
C13	0.69057 (13)	0.85257 (11)	−0.07591 (8)	0.0227 (3)	
H13	0.6378	0.7849	−0.0639	0.027*	
C14	0.73616 (14)	0.86444 (11)	−0.15246 (8)	0.0251 (3)	
H14	0.7157	0.8048	−0.1925	0.030*	
C15	0.81319 (14)	0.96438 (11)	−0.17238 (8)	0.0258 (3)	
H15	0.8441	0.9724	−0.2257	0.031*	
C16	0.84296 (13)	1.04920 (10)	−0.11474 (8)	0.0240 (3)	
H16	0.8941	1.1167	−0.1285	0.029*	
C17	0.70253 (13)	0.71503 (10)	0.09513 (7)	0.0183 (2)	
C18	0.65617 (14)	0.61097 (10)	0.05873 (7)	0.0232 (3)	
H18	0.5536	0.6029	0.0296	0.028*	
C19	0.75686 (14)	0.51809 (11)	0.06393 (8)	0.0253 (3)	
H19	0.7220	0.4476	0.0385	0.030*	
C20	0.90795 (14)	0.52715 (10)	0.10585 (8)	0.0230 (3)	
C21	0.95452 (14)	0.63162 (11)	0.14195 (8)	0.0239 (3)	
H21	1.0576	0.6403	0.1702	0.029*	
C22	0.85309 (14)	0.72368 (10)	0.13750 (8)	0.0218 (3)	
H22	0.8872	0.7938	0.1639	0.026*	
C23	1.01741 (15)	0.42637 (11)	0.11254 (9)	0.0319 (3)	
H23A	1.1244	0.4540	0.1171	0.038*	
H23B	0.9930	0.3783	0.0628	0.038*	
H23C	1.0059	0.3813	0.1621	0.038*	
H1	0.6867 (18)	1.0689 (14)	0.2399 (10)	0.045 (5)*	

### Atomic displacement parameters (Å^2^)

**Table:**

	*U*^11^	*U*^22^	*U*^33^	*U*^12^	*U*^13^	*U*^23^
O1	0.0220 (4)	0.0226 (5)	0.0270 (5)	−0.0030 (3)	0.0040 (4)	0.0034 (3)
N1	0.0233 (5)	0.0206 (5)	0.0197 (5)	−0.0025 (4)	0.0054 (4)	−0.0022 (4)
C1	0.0162 (5)	0.0199 (6)	0.0208 (6)	0.0016 (4)	0.0033 (5)	0.0012 (5)
C2	0.0160 (5)	0.0189 (6)	0.0192 (6)	0.0008 (4)	0.0023 (4)	0.0015 (4)
C3	0.0175 (5)	0.0181 (6)	0.0185 (6)	−0.0013 (4)	0.0024 (5)	0.0007 (4)
C4	0.0171 (5)	0.0194 (6)	0.0208 (6)	0.0011 (4)	0.0034 (5)	0.0026 (5)
C5	0.0175 (5)	0.0212 (6)	0.0208 (6)	0.0023 (4)	0.0033 (5)	0.0019 (5)
C6	0.0252 (6)	0.0265 (7)	0.0209 (7)	−0.0007 (5)	0.0075 (5)	0.0002 (5)
C7	0.0220 (6)	0.0233 (6)	0.0237 (7)	0.0015 (4)	0.0066 (5)	0.0044 (5)
C8	0.0176 (5)	0.0199 (6)	0.0232 (6)	0.0032 (4)	0.0019 (5)	0.0058 (5)
C9	0.0206 (6)	0.0179 (6)	0.0262 (7)	0.0000 (4)	0.0036 (5)	−0.0021 (5)
C10	0.0205 (6)	0.0177 (6)	0.0313 (7)	−0.0003 (4)	0.0071 (5)	0.0033 (5)
C11	0.0170 (5)	0.0198 (6)	0.0233 (6)	0.0030 (4)	0.0037 (5)	0.0044 (5)
C12	0.0162 (5)	0.0204 (6)	0.0196 (6)	0.0025 (4)	0.0023 (5)	0.0030 (4)
C13	0.0222 (6)	0.0237 (6)	0.0217 (6)	−0.0018 (5)	0.0015 (5)	0.0007 (5)
C14	0.0271 (6)	0.0273 (6)	0.0202 (6)	0.0007 (5)	0.0020 (5)	−0.0009 (5)
C15	0.0257 (6)	0.0310 (7)	0.0221 (7)	0.0059 (5)	0.0077 (5)	0.0059 (5)
C16	0.0232 (6)	0.0238 (6)	0.0264 (7)	0.0029 (5)	0.0086 (5)	0.0072 (5)
C17	0.0195 (5)	0.0201 (6)	0.0163 (6)	−0.0023 (4)	0.0060 (5)	0.0015 (4)
C18	0.0199 (6)	0.0244 (6)	0.0249 (7)	−0.0032 (5)	0.0020 (5)	−0.0024 (5)
C19	0.0270 (6)	0.0213 (6)	0.0284 (7)	−0.0025 (5)	0.0066 (5)	−0.0043 (5)
C20	0.0239 (6)	0.0240 (6)	0.0228 (6)	0.0016 (5)	0.0090 (5)	0.0013 (5)
C21	0.0189 (6)	0.0261 (6)	0.0261 (7)	−0.0015 (5)	0.0016 (5)	0.0030 (5)
C22	0.0225 (6)	0.0208 (6)	0.0222 (6)	−0.0029 (4)	0.0033 (5)	−0.0011 (5)
C23	0.0312 (7)	0.0278 (7)	0.0376 (8)	0.0055 (5)	0.0081 (6)	−0.0010 (6)

### Geometric parameters (Å, º)

**Table:**

O1—C8	1.2366 (14)	C11—C16	1.4134 (17)
N1—C5	1.3454 (15)	C11—C12	1.4245 (15)
N1—C1	1.4039 (16)	C12—C13	1.4163 (17)
N1—H1	0.902 (17)	C13—C14	1.3700 (17)
C1—C2	1.3849 (16)	C13—H13	0.9500
C1—C9	1.4156 (15)	C14—C15	1.4097 (17)
C2—C12	1.4318 (16)	C14—H14	0.9500
C2—C3	1.5338 (15)	C15—C16	1.3601 (18)
C3—C4	1.5049 (16)	C15—H15	0.9500
C3—C17	1.5301 (15)	C16—H16	0.9500
C3—H3	1.0000	C17—C18	1.3844 (16)
C4—C5	1.3571 (16)	C17—C22	1.3889 (17)
C4—C8	1.4360 (16)	C18—C19	1.3904 (16)
C5—C6	1.5002 (16)	C18—H18	0.9500
C6—C7	1.5308 (16)	C19—C20	1.3908 (18)
C6—H6A	0.9900	C19—H19	0.9500
C6—H6B	0.9900	C20—C21	1.3872 (17)
C7—C8	1.5257 (17)	C20—C23	1.5093 (16)
C7—H7A	0.9900	C21—C22	1.3876 (16)
C7—H7B	0.9900	C21—H21	0.9500
C9—C10	1.3571 (17)	C22—H22	0.9500
C9—H9	0.9500	C23—H23A	0.9800
C10—C11	1.4158 (17)	C23—H23B	0.9800
C10—H10	0.9500	C23—H23C	0.9800
			
C5—N1—C1	119.46 (10)	C16—C11—C10	121.29 (11)
C5—N1—H1	115.4 (10)	C16—C11—C12	119.57 (11)
C1—N1—H1	123.1 (10)	C10—C11—C12	119.11 (11)
C2—C1—N1	121.12 (10)	C13—C12—C11	117.47 (11)
C2—C1—C9	121.35 (11)	C13—C12—C2	122.52 (11)
N1—C1—C9	117.52 (10)	C11—C12—C2	120.01 (11)
C1—C2—C12	118.24 (10)	C14—C13—C12	121.50 (11)
C1—C2—C3	121.76 (10)	C14—C13—H13	119.2
C12—C2—C3	119.95 (10)	C12—C13—H13	119.2
C4—C3—C17	111.17 (9)	C13—C14—C15	120.54 (12)
C4—C3—C2	109.12 (9)	C13—C14—H14	119.7
C17—C3—C2	111.26 (9)	C15—C14—H14	119.7
C4—C3—H3	108.4	C16—C15—C14	119.50 (12)
C17—C3—H3	108.4	C16—C15—H15	120.3
C2—C3—H3	108.4	C14—C15—H15	120.3
C5—C4—C8	108.82 (11)	C15—C16—C11	121.41 (11)
C5—C4—C3	123.27 (10)	C15—C16—H16	119.3
C8—C4—C3	127.91 (11)	C11—C16—H16	119.3
N1—C5—C4	123.07 (11)	C18—C17—C22	117.82 (11)
N1—C5—C6	122.75 (11)	C18—C17—C3	122.06 (10)
C4—C5—C6	114.18 (11)	C22—C17—C3	120.12 (10)
C5—C6—C7	102.61 (10)	C17—C18—C19	121.28 (11)
C5—C6—H6A	111.2	C17—C18—H18	119.4
C7—C6—H6A	111.2	C19—C18—H18	119.4
C5—C6—H6B	111.2	C18—C19—C20	120.83 (11)
C7—C6—H6B	111.2	C18—C19—H19	119.6
H6A—C6—H6B	109.2	C20—C19—H19	119.6
C8—C7—C6	105.69 (9)	C21—C20—C19	117.84 (11)
C8—C7—H7A	110.6	C21—C20—C23	121.01 (11)
C6—C7—H7A	110.6	C19—C20—C23	121.15 (11)
C8—C7—H7B	110.6	C20—C21—C22	121.15 (11)
C6—C7—H7B	110.6	C20—C21—H21	119.4
H7A—C7—H7B	108.7	C22—C21—H21	119.4
O1—C8—C4	127.77 (12)	C21—C22—C17	121.07 (11)
O1—C8—C7	123.64 (10)	C21—C22—H22	119.5
C4—C8—C7	108.59 (10)	C17—C22—H22	119.5
C10—C9—C1	120.71 (11)	C20—C23—H23A	109.5
C10—C9—H9	119.6	C20—C23—H23B	109.5
C1—C9—H9	119.6	H23A—C23—H23B	109.5
C9—C10—C11	120.54 (11)	C20—C23—H23C	109.5
C9—C10—H10	119.7	H23A—C23—H23C	109.5
C11—C10—H10	119.7	H23B—C23—H23C	109.5
			
C5—N1—C1—C2	−8.76 (16)	C9—C10—C11—C16	−178.39 (11)
C5—N1—C1—C9	170.95 (10)	C9—C10—C11—C12	−0.31 (17)
N1—C1—C2—C12	179.22 (10)	C16—C11—C12—C13	0.79 (16)
C9—C1—C2—C12	−0.47 (16)	C10—C11—C12—C13	−177.32 (10)
N1—C1—C2—C3	−3.34 (16)	C16—C11—C12—C2	179.77 (10)
C9—C1—C2—C3	176.96 (10)	C10—C11—C12—C2	1.65 (16)
C1—C2—C3—C4	13.56 (14)	C1—C2—C12—C13	177.68 (10)
C12—C2—C3—C4	−169.04 (10)	C3—C2—C12—C13	0.19 (16)
C1—C2—C3—C17	−109.46 (12)	C1—C2—C12—C11	−1.24 (16)
C12—C2—C3—C17	67.93 (13)	C3—C2—C12—C11	−178.73 (9)
C17—C3—C4—C5	109.07 (12)	C11—C12—C13—C14	0.08 (17)
C2—C3—C4—C5	−14.00 (15)	C2—C12—C13—C14	−178.86 (11)
C17—C3—C4—C8	−70.90 (14)	C12—C13—C14—C15	−0.67 (18)
C2—C3—C4—C8	166.03 (11)	C13—C14—C15—C16	0.35 (18)
C1—N1—C5—C4	8.74 (17)	C14—C15—C16—C11	0.55 (18)
C1—N1—C5—C6	−170.53 (10)	C10—C11—C16—C15	176.95 (11)
C8—C4—C5—N1	−176.27 (10)	C12—C11—C16—C15	−1.12 (17)
C3—C4—C5—N1	3.75 (17)	C4—C3—C17—C18	103.97 (13)
C8—C4—C5—C6	3.06 (14)	C2—C3—C17—C18	−134.18 (11)
C3—C4—C5—C6	−176.92 (10)	C4—C3—C17—C22	−76.38 (13)
N1—C5—C6—C7	175.76 (10)	C2—C3—C17—C22	45.46 (15)
C4—C5—C6—C7	−3.57 (13)	C22—C17—C18—C19	−0.29 (18)
C5—C6—C7—C8	2.56 (12)	C3—C17—C18—C19	179.36 (11)
C5—C4—C8—O1	178.03 (11)	C17—C18—C19—C20	−0.19 (19)
C3—C4—C8—O1	−2.0 (2)	C18—C19—C20—C21	−0.13 (19)
C5—C4—C8—C7	−1.15 (13)	C18—C19—C20—C23	179.35 (12)
C3—C4—C8—C7	178.82 (11)	C19—C20—C21—C22	0.94 (18)
C6—C7—C8—O1	179.74 (11)	C23—C20—C21—C22	−178.54 (11)
C6—C7—C8—C4	−1.04 (12)	C20—C21—C22—C17	−1.46 (19)
C2—C1—C9—C10	1.84 (17)	C18—C17—C22—C21	1.10 (18)
N1—C1—C9—C10	−177.87 (10)	C3—C17—C22—C21	−178.56 (11)
C1—C9—C10—C11	−1.41 (17)		

### Hydrogen-bond geometry (Å, º)

Cg1 is the centroid of the C17–C22 ring.

**Table:**

*D*—H···*A*	*D*—H	H···*A*	*D*···*A*	*D*—H···*A*
N1—H1···O1^i^	0.902 (17)	1.983 (17)	2.8519 (13)	161.2 (14)
C15—H15···*Cg*1^ii^	0.95	3.00	3.8110 (15)	145

Symmetry codes: (i) −*x*+1, *y*+1/2, −*z*+1/2; (ii) −*x*, −*y*+2, −*z*.
